# Attitudes to Short-Term Staffing and Workforce Priorities of Community Users of Remote Aboriginal Community-Controlled Health Services: A Qualitative Study

**DOI:** 10.3390/ijerph21040482

**Published:** 2024-04-15

**Authors:** Zania Liddle, Michelle S. Fitts, Lisa Bourke, Lorna Murakami-Gold, Narelle Campbell, Deborah J. Russell, Supriya Mathew, Jason Bonson, Edward Mulholland, John S. Humphreys, Yuejen Zhao, John Boffa, Mark Ramjan, Annie Tangey, Rosalie Schultz, John Wakerman

**Affiliations:** 1 Menzies School of Health Research, Charles Darwin University, Alice Springs, NT 0870, Australia; zania.liddle@menzies.edu.au (Z.L.); deborah.russell@menzies.edu.au (D.J.R.); supriya.mathew@menzies.edu.au (S.M.); jason.bonson@flinders.edu.au (J.B.); john.wakerman@menzies.edu.au (J.W.); 2 Department of Rural Health, The University of Melbourne, Shepparton, VIC 3630, Australia; bourke@unimelb.edu.au; 3 Poche SA and NT, College of Medicine and Public Health, Flinders University, Alice Springs, NT 0870, Australia; lorna.murakamigold@flinders.edu.au; 4 College of Medicine and Public Health, Flinders University, Darwin, NT 0800, Australia; narelle.campbell@flinders.edu.au; 5 Miwatj Health Aboriginal Corporation, Nhulunbuy, NT 0880, Australia; eddie.mulholland@outlook.com; 6 School of Rural Health, Monash University, Bendigo, VIC 3550, Australia; john.humphreys@monash.edu; 7 Northern Territory Department of Health, Northern Territory Government, Darwin, NT 0800, Australia; yuejen.zhao@nt.gov.au; 8 Central Australian Aboriginal Congress, Alice Springs, NT 0870, Australia; john.boffa@caac.org.au; 9 Top End Health Service, Northern Territory Government, Casuarina, NT 0810, Australia; mark.ramjan@nt.gov.au; 10 Ngaanyatjarra Health Service, Alice Springs, NT 0870, Australia; annie.tangey@nghealth.org.au (A.T.); rosalie.schultz@nghealth.org.au (R.S.)

**Keywords:** Aboriginal and Torres Strait Islander, primary health, workforce, remote, Australia

## Abstract

In recent years, there has been an increasing trend of short-term staffing in remote health services, including Aboriginal Community-Controlled Health Services (ACCHSs). This paper explores the perceptions of clinic users’ experiences at their local clinic and how short-term staffing impacts the quality of service, acceptability, cultural safety, and continuity of care in ACCHSs in remote communities. Using purposeful and convenience sampling, community users (aged 18+) of the eleven partnering ACCHSs were invited to provide feedback about their experiences through an interview or focus group. Between February 2020 and October 2021, 331 participants from the Northern Territory and Western Australia were recruited to participate in the study. Audio recordings were transcribed verbatim, and written notes and transcriptions were analysed deductively. Overall, community users felt that their ACCHS provided comprehensive healthcare that was responsive to their health needs and was delivered by well-trained staff. In general, community users expressed concern over the high turnover of staff. Recognising the challenges of attracting and retaining staff in remote Australia, community users were accepting of rotation and job-sharing arrangements, whereby staff return periodically to the same community, as this facilitated trusting relationships. Increased support for local employment pathways, the use of interpreters to enhance communication with healthcare services, and services for men delivered by men were priorities for clinic users.

## 1. Introduction

Improving access to remote primary healthcare (PHC) services is linked to addressing disparities between Aboriginal and Torres Strait Islander peoples (with respect herein referred to as Aboriginal) and other Australians [1,2]. One barrier to ‘closing the gap’ between Aboriginal and non-Aboriginal health is the limited access to PHC services, underpinned by difficulties with recruitment of appropriately trained and culturally competent staff, as well as an unstable health workforce [3]. There are a number of key factors influencing the high nursing turnover in remote areas including social and geographical isolation, high workload, extended scope of practice, exhaustion and burnout, as well as poor housing and infrastructure issues [4,5,6,7,8,9,10]. Climate change is also exacerbating healthcare shortages for underserved populations, with rising temperatures contributing to health staff reconsidering working in remote areas of Australia [11]. There are also a range of factors associated with the recruitment and retention of healthcare staff to remote areas including being of rural origin, completion of training in a rural location, the lifestyle that is offered in remote areas, playing a role in improving Aboriginal and Torres Strait Islander health, and adequate access to resources and support for the family of the health professional (e.g., childcare, education for children) [12,13,14,15]. Anecdotally, there is a commonly held belief that there is a relationship between continuity of care and a stable workforce, but there is little evidence to support this, as is demonstrated by Jones and colleagues [16]. Securing a health workforce to provide culturally appropriate and evidence-based, clinically sound care for Aboriginal peoples in remote regions has been difficult [17]. At the core of Aboriginal community control of healthcare is the provision of culturally appropriate and holistic health services, ensuring self-reliance, self-determination, and appropriate and acceptable healthcare, as laid out in articles of the United Nations Declaration on the Rights of Indigenous Peoples [18].

In response to health inequalities and the failure of mainstream services to provide adequate healthcare, the first Aboriginal Community-Controlled Health Services (ACCHSs) were established as advocacy services from the early 1970s [19,20]. ACCHSs aim to provide PHC services for their clients which are culturally appropriate, autonomous, and community-governed, where local Aboriginal communities are responsible for initiating, planning, and governing those services [21,22,23]. Several studies have found that compared to mainstream services, ACCHSs are more frequently accessed by Aboriginal peoples and perform better in areas of best practice care, monitoring clinical performance, increasing engagement of Aboriginal community members, Aboriginal employment, and better leadership in training non-Indigenous staff in Aboriginal health matters [24,25,26,27]. Fulfilling the aspirations of Aboriginal communities, there are a growing number of examples of government-run PHC clinics that are transitioning to community-controlled healthcare [28,29].

One of the current challenges for ACCHSs delivering health services is workforce shortages due to maldistribution and a high staff turnover. As a result, these remote services have increasingly relied on short-term, fly in/fly out health professionals. These health professionals are usually employed on a temporary basis (e.g., locum tenens or agency nurses), mainly through health professional recruitment agencies. Short-term workforce arrangements create challenges, including difficulties in building relationships with clinic users and providing continuity of care, high costs, and the challenges of ensuring consistently culturally safe and secure care [3]. While much has been written about the experiences of the remote health workforce [4,5,6,7,8,9] and some research relating to other pertinent health service issues exists, very little has been previously reported from the perspectives of health service users in remote communities. This paper aims to (i) document and better understand the experiences for community users in relation to short-term staff in their local clinic; (ii) understand the impacts of a high staff turnover for community users; and (iii) provide ACCHSs with evidence-based recommendations of community priorities and preferences to inform the development of policy and strategy relating to staff recruitment and retention.

## 2. Materials and Methods

### 2.1. Leadership

The methods used for data collection were designed by Aboriginal and non-Aboriginal investigators. Leadership of the project was complemented by a Steering Committee, consisting of members representing each of the partner ACCHSs involved. The Steering Committee provided direction and advice to the project relating to participant recruitment, navigating ACCHSs, development of the interview process and instrument, advice on data collection, accessing relevant resources (such as interpreters), as well as reporting the findings back to communities and service staff.

### 2.2. Ethics

The study received ethics approval from the Human Research Ethics Committee of the Northern Territory Department of Health and Menzies School of Health Research (project number DR03171), the Central Australian Human Research Ethics Committee (CA-19-3493), and the Western Australian Aboriginal Health Ethics Committee (WAAHEC-938). The study complied with national guidelines for research with Aboriginal and Torres Strait Islander peoples [30].

### 2.3. Setting

Most of the communities involved in the study were in very remote locations in the Northern Territory (NT) and Western Australia (WA) [31]. Access to these isolated communities required several hours of travel on unsealed roads, with communities up to 800 km from the nearest regional centre. Populations in these very remote communities ranged from approximately 100 to 3000. Clinics in these communities varied from large facilities with multiple consulting rooms, a relatively large staff, and opening hours from Mondays to Fridays with 24/7 on call services, to small facilities with no more than two consulting rooms and two staff members. A small number of clinics operated on a part-time basis only, and some were closed over this period of time, when human resources were scarce and where staffing shortages meant that resources needed to be relocated to larger clinics (for more information refer to Fitts et al., 2021) [32].

### 2.4. Participant Eligibility

Community members could take part if they were aged 18 years or older and had accessed any of the services provided by their local clinic in the community in which they were currently living or clinics where the services accessed were other locations of partner ACCHSs. There were no criteria for when participants had to have last used their local clinic and the frequency at which participants accessed services. The research team engaged with community users who accessed their local clinic for various reasons, including for general practitioner (GP) services, health screenings, management of chronic illnesses and education. Clinic users also accessed their local clinic for support with new babies, child health, women’s health, men’s health, as well as medical retrieval, outreach, dental, podiatry, and other specialist and allied health services. Community users described specialist and allied health as those services that were not GP/everyday healthcare and were provided either in the community or in regional centres. Terms such as women’s doctor/nurse, ‘kid’ doctor, foot doctor, diabetes nurse, and heart doctor were used by community users. While the terms patient, clinic user, and community user are used interchangeably in workforce research, in this study, we refer to community members who attend a local ACCHS clinic as ‘community users’.

### 2.5. Participant Recruitment

A team of two researchers working alongside a community-based researcher visited each remote community to conduct interviews and focus groups with community users. As there were multiple community clinics involved, each of the participating ACCHSs identified which of their communities were the most suitable for recruitment sites and available for participant recruitment. Drawing on advice from the leadership of partnering ACCHSs, communities that were visited included those with clinics with stable staffing, as well as those with a high staff turnover.

Prior to each visit, the research team liaised with the local clinic manager, as well as other service providers, community groups, and key leaders in the community. While community users were one of several participant groups in the study [32], this paper only focuses on community users of the partnering ACCHSs.

As ACCHSs aim to provide a broad range of services, the research team promoted the project to a variety of local agencies in order to optimise the participation of community users. Examples of these agencies included (i) ranger groups and art centres; (ii) groups run by existing services such as men’s and women’s groups; and (iii) through the remote clinics that were serviced by the partnering ACCHSs. The project was also promoted in public spaces, including shopping centres in regional locations, outside remote community stores, as well as at events such as National Aborigines and Islanders Day Observance Committee (NAIDOC) week.

### 2.6. Data Collection

Data collection took place between February 2020 and October 2021 across twenty locations, mainly very remote [31]. Community users were invited to take part in an interview or small group discussion according to individual preference. Information about the project and participant rights were explained to all before proceeding with the interview or discussion group. In addition, participants were provided with a copy of the project flipchart, information statement, and consent form. Participants had the option to provide verbal or written consent, and the opportunity to ask questions about any aspect of the project was available to participants.

As the participants were almost always multilingual, where possible, the research team worked with interpreters from the NT Aboriginal Interpreter Service, as well as locally sourced interpreters and community-based researchers. In addition, interpreters and community-based researchers assisted with supporting the research team to navigate around communities, as well as providing advice on the best ways to engage with community structures and individuals. Critically, these data were collected during the COVID-19 pandemic, when workforce shortages were greatly exacerbated. During the acute phase of the pandemic, travel across jurisdictional borders and to remote communities by short-term staff was restricted and healthcare workers were needed for immunisation programs and quarantine facilities. Many staff were unable to work due to illness and there was a key focus on the prevention of transmission of COVID-19 to community users, which required the redeployment of staff from their normal duties [33].

Informed by a program logic model [34,35,36], topics covered in community user interviews and discussion groups included the following: what users liked about ACCHS services, particularly about what works and what healthcare services they utilise; what impact short-term staffing or rotating staff have on the delivery of the health services that they receive; the governance and management of the service in the context of community-controlled health; and the impacts of COVID-19 on the community and their access to PHC. For most of the data collection, two research team members conducted the focus group discussions and interviews. Most community users preferred a focus group discussion, with only a small number of interviews being completed with individual community users. The size of focus groups ranged from two to eight participants. Most of the focus group discussions and interviews were recorded using written notes, with a small number audio-recorded. The research team conferred at the end of each focus group or interview, making notes and seeking clarification from participants if this was needed. To maintain a culturally safe environment, one of the focus group discussions with male community users was completed by two local male health service staff members following briefing by the research team. Upon completion of those discussions, the male health service staff members briefed the research team on the feedback from male community users. Other focus group discussions with men were conducted by a male research team member.

### 2.7. Data Analysis

Audio recordings were transcribed verbatim into separate Word documents by a professional transcriber and then checked by one of the research team members for accuracy against the original recording. Written notes were also transcribed into a typed Word format. The research team member assigned a unique identifier to each transcript. The unique identifier was retained, with information about participants and their location identity removed. All transcripts from focus group interviews were imported into the qualitative data analysis software NVivo v12 software (Denver, CO, USA).

The two researchers who carried out the majority of the fieldwork (ZL and MF) deductively analysed the data by coding the data into similar category codes [37]. Patterns in the codes and links between the codes were identified and used to organise the data into meaningful themes linked to the project aims, which related to short-term staffing and workforce impacts; community control; what works; what does not work; cultivating an appropriate workforce; and COVID-19 impacts and lessons learned. Initial themes and sub-themes were developed independently by the two research team members and subsequently discussed to clarify minor points and reach agreement on the labelling of the themes. The labelling of themes and clarification of sub-themes was continuously reviewed throughout the analysis. Integral to applying an inclusive lens to the coding and data analysis process and reaching common understandings about the data was leadership from a broader group of authors (LB, LMG, and NC). In the main, this leadership took place as monthly scheduled meetings over the period of data analysis.

## 3. Results

Overall, 331 community users, of whom 315 (95%) identified as Aboriginal and/or Torres Strait Islander, participated in an interview or focus group discussion. In very isolated communities, some ACCHSs offer services to non-Indigenous people. Therefore, a small number of non-Indigenous community members were also interviewed. The analysis of the community user interview and focus group discussion data revealed a range of different elements of service access and delivery that work well for community users and others that impacted their ability to access health services and/or their experiences of using these services. Community users had important perceptions about what characterised the ‘right’ staff to support their access to PHC. Suggestions were also made by community users about cultivating an appropriate workforce for remote PHC delivery to enable better access, as well as delivery of services, by their local clinic.

### 3.1. ‘What Works’ and Supports a Good Community User Experience

Across all ACCHSs, the common elements that support positive community user experiences relate to the access to services, cultural safety practices, quality of care, and effective communications, as well as a holistic healthcare approach.

#### 3.1.1. Access to Services

Many community users reported that they have access to a comprehensive range of quality PHC services, provided by health professionals “*who do a good job*”. Amenities outside of the PHC services that were described as helping to support community users’ health and wellbeing included access to laundry facilities to wash clothing; bathroom facilities to have a shower; and kitchen facilities to cook food. Supported activities such as traditional bush medicine collection, as well other services including drug and alcohol services, allied health services, and sport and recreational services, were also reported to support community users’ health and wellbeing.

#### 3.1.2. Quality Care and Effective Communications

In general, community users engaged with a diverse number of healthcare workers providing services at their local clinic, including Aboriginal Health Practitioners (AHPs), GPs, Remote Area Nurses (RANs), diabetes educators, child and maternity health nurses, women’s health, podiatry and dental services, as well as other visiting specialist and allied health practitioner services:

*Medication. Eye check, foot doctor, ears check. They had a truck here last week that was doing ears and eyes, health promotion.*

(ACCHS #1)

*Drs and Nurses encourage me to walk around following having a stroke*

(ACCHS #2)

Community users from all the communities that were visited valued having access to visiting specialist and allied health teams in their communities, as this meant that they did not have to leave their local community to access these services and care. Many participants also suggested more dialysis chairs in some communities, so that community members do not have to move away from kin and country to regional centres to access dialysis.

#### 3.1.3. Holistic Healthcare

Community users valued the breadth of holistic healthcare services that were available through their local clinic, as well as how they were looked after by ACCHSs. ACCHSs provided a range of services outside of traditional PHC including bush medicine, laundry, ablution, and kitchen facilities:

*[name for people sleeping rough] they have a shower, make tea and coffee. They see them while they’re doing that. The staff at the clinic, they see them while they are cleaning clothes, making tea and coffee.*

(ACCHS #6)

*We’ve got our own bush medicine. Sometimes we use our own medicine and sometimes we use [local name for non-Indigenous] medicine. Our own bush medicine is very important to [Aboriginal] people.*

(ACCHS #3)

*Community controlled, it’s got a bit more of, it seems to have a bit more affinity. And I don’t know whether it’s got more funds or not, but they certainly throw a lot more at you. […] Yeah, in terms of … making sure everyone’s looked after.*

(ACCHS #5)

Community users from some ACCHSs suggested that clinics should have more mental health supports readily available in communities, including youth mental health services and drug and alcohol services.

#### 3.1.4. Culturally Safe Practice

Cultural safety in the context of health service delivery is defined as the way patients feel, as well as how they are treated by health professionals as they navigate the health space [38]. Culturally appropriate practices are evident in the community user data, demonstrated by culturally sensitive and effective communications, including the use of interpreters and translators of the local language; non-Aboriginal staff learning the local language; gender-specific services for men and women; and involvement of local Aboriginal people in service delivery as well as in non-clinical roles:

*The clinic is good cultural way.*

(ACCHS #2)

Community users from five ACCHSs identified practices that were currently in place and had a variety of suggestions for ways to improve local clinic systems. These included practices in order to further strengthen culturally safe practice, making the clinic a place where community users felt culturally safe and experienced a sense of belonging:

*Men’s clinic—there’s no fear factor. Very comfortable environment. I’m confident there. Used to be at an old clinic, now new clinic. Has coffee, tea, sugar, guitar, lots of visual things, TV, Aboriginal Health messages, dart board.*

(ACCHS #6)

*They do teach some of the staff words in language.*

(ACCHS #5)

### 3.2. ‘The Right Staff’

Community users across all ACCHSs spoke about the qualities of staff that contribute towards a strong relationship between healthcare worker and patient and what qualities contributed to community users not accessing the clinic. Sub-themes under the right staff include the importance of trusting relationships, the qualities and skills of staff, mutual respect, and effective communication.

#### 3.2.1. Importance of Trusting Relationships

Community users from all ACCHSs reported a link between the opportunity to build strong and trusting therapeutic relationships and staff who stay for a long time:

*Long term nurses get to know us, [We] get to know them.*

(ACCHS #4)

Long-term staff (over two years) were regarded by community users as being invested in the community and were more aware of the medical histories of community users and their families. Some community users have established relationships with long-term clinic staff and sought to see these staff members wherever possible. Some community users talked about good, strong relationships with GPs and RANs who worked at the clinics long-term, even if the staff were working at their clinic on a rotating basis, that is, periodically leaving the service and subsequently returning to the same community.

Staff turnover had an impact on community users’ ability to build trusting relationships with healthcare workers and to seek help for all their health needs:

*We don’t trust them when they change.*

(ACCHS #4)

Another commented:

*It does have a detrimental effect, you know, on people’s health in that they’re sick of telling the same stories, so half the time they only tell half the story, by the time issues come up, then you know, it has a big health impact.*

(ACCHS #2)

Aware that the clinic often had new RANs who stayed for a short time, typically six weeks, some community users were frustrated that they were repeatedly asked the same questions, thus prolonging their consultations:

*Every time you go there [the clinic] you see a different person, so then you’ve got to tell your story again, or your history, and plus, you know, you get comfortable with one and then you know, then you’ve got to talk to another.*

(ACCHS #2)

Some community users mentioned that staff needed to be better prepared by familiarising themselves with patient information from the health service records prior to their consultation.

#### 3.2.2. Qualities and Skills of Staff

Community users described staff turnover as a longstanding issue for their local health service. Community users held a desire for health staff to have advanced clinical skills and apply culturally safe practices, along with respectful and effective communications being important. Furthermore, a demonstration of real commitment to stay and work in their community long-term is preferred and valued:

*[We want] someone who wants to stay. They have to want to stay, you can’t come to somewhere so remote, um, and two years isn’t staying, like that’s not long enough.*

(ACCHS #5)

*Generally, prefer to see usual doctors who are mostly the dedicated ones and quality of service from these doctors is good.*

(ACCHS #1)

Community users recognised that staff at ACCHSs need to have good personal health to work in remote communities. Some community users mentioned that many non-local healthcare workers who come to work in ACCHSs are older and have their own health conditions, and this impacted on community users’ access to healthcare. One example shared was of a health professional with a hearing impairment and a worry by the community user that this could impact upon patient safety and confidentiality:

*The whole of the clinic knows your business because he can’t [hear], you’ve got to talk really loud. Oh yeah, okay. You know? And it sort of makes people uncomfortable because obviously it’s their private business and they don’t want, you know, everyone else around them to be knowing what they’re talking about.*

(ACCHS #2)

#### 3.2.3. Mutual Respect and Communication

Community users reported that they were more likely to utilise health services if they felt welcome at the clinic. When asked about what ‘good staff’ were, qualities mentioned included kindness and caring, having good listening skills, and mutual respect for Aboriginal peoples and culture. Community users from one ACCHS spoke about previous RANs who lacked respect and spoke ‘rough’ to community members:

*They [clinic staff] should be able to work in the community in a good way. Feeling the same manymak [good]. There are ‘no good’ staff sometime—they like a doctor, he talk really roughly for [Aboriginal people] and he treated people the wrong way.*

(ACCHS #3)

*She [a nurse] was cranky, angry. Everybody didn’t like her. They couldn’t speak with her at the clinic.*

(ACCHS #3)

It was important to community users that unsuitable staff be identified and managed by the health service early. Proactive management might include new staff receiving an appropriate introduction to the local community and relevant training and professional development. In one instance, community users told us that the land permit of a healthcare worker had been revoked by the community because of disrespectful behaviour towards clinic clients.

### 3.3. What Impacts Community Users’ Experiences Accessing Their Local Clinic

Although there were a number of qualities and elements that community users appreciated and liked about their local clinic, there were specific experiences that had a negative impact on their access to the clinic. These include wait times, patient ‘worries’, access to specialist and allied health services, and transport. The term ‘worries’ was frequently used in conversation with community users to explain the diversity of concerns that impact their access to clinic services.

#### 3.3.1. Wait Times

Some community users said it was easy to make an appointment at the clinic and the wait time to see a staff member was ‘not long’ (around 20 min). For others however, the wait time was too long (explained as over an hour). In some examples where the clinic was busy, community users were directed to the local hospital. Some community users reported leaving the clinic without having their health needs addressed because the wait time was so long. Wait times were also an issue for community users who worked, as they were unable to wait for extended periods of time because of work commitments:

*If we are going to go, if we have an appointment at the clinic, we need to get check-up straight away. We can’t wait for 2–3 h because we got a big job to do here [place of work].*

(ACCHS #1)

*If they are busy, they tell you to go [to] the hospital.*

(ACCHS #2)

*Sometimes wait time is too long. Some people just go back home [do not see a healthcare worker].*

(ACCHS #6)

When asked about why extended wait times could be experienced, some community users attributed the long wait time to the clinic being short-staffed, as well as the completion of health checks (which require a longer consultation):

*Waiting time—big, sometimes we sit there long time, 5 to 6 h. Not enough staff.*

(ACCHS #3)

*When we go to [name of the clinic] we have to wait so long. Sometimes they are telling a story in the clinic and forgetting us. They are not looking after us. They are telling us they only got one or two lady [nurse].*

(ACCHS #3)

Long wait times for after-hours healthcare, as well as an absence of a response or an explanation about why wait times were long, was also reported in some communities:

*There is a button at the front so we can talk to them [clinic staff] out the front first but they don’t always pick up.*

(ACCHS #4)

#### 3.3.2. Access to Specialists and Allied Health Services

Community users valued access to GPs and visiting specialists and allied health teams, as this meant that they did not have to leave their community to access healthcare. Community users in two communities reported that there was a lack of reliable information about these visits, with wider promotion needed (e.g., dissemination of information to other agencies in the community and on social media). To achieve this, suggestions from the community users included the display of posters at the local store and on the local clinic board to inform of upcoming visits. Other community users suggested that information about GP and visiting specialist and allied health team visits could be circulated to workplaces ahead of time, allowing community users time to make arrangements with their workplaces. Communities involved in the project had differing access to GPs and visiting specialist and allied health teams. The need for longer visits by specialist and allied health teams was expressed by community users in communities where there were fewer regular visits from GPs and visiting specialist and allied health teams:

*I think they [the dentist] come once a month but not sure…. it was two months since they last came out. They seem to wait to see people until the tooth is fully infected and has to be removed.*

(ACCHS #4)

*We haven’t had an optometrist since 2019. Communication, get it out to the people. People don’t know that they’re around. We haven’t had a dentist for 12 months.*

(ACCHS #5)

*Stay a bit longer, specialists. Physio is here for a day or two. Keep missing the specialist because of work. I have never been getting notices for the specialist teams until I asked for them.*

(ACCHS #5)

*What if we can have a dentist here [based in community]? We have to wait 2–3 months. That’s the main reason I didn’t like about this clinic. The pain keep[s] coming back and I have to wait until next time.*

(ACCHS #3)

#### 3.3.3. Patient ‘Worries’

As patients, community users had a range of ‘worries’, including a poor quality of service, language difficulties and poor communication, a high staff turnover, and clinicians struggling to accurately diagnose and manage patients with complex conditions.

Some community users reported that healthcare workers did not provide adequate information, nor allow enough time to explain a patient’s medical condition and treatment. Further worries included them being either too quick or too slow to make a diagnosis or commence treatment, and/or that they were not attuned to the needs of the patient or his/her family. Community users reported being confused and stressed when they receive different treatment advice and diagnoses from different healthcare workers, as well as when they do not get the treatments that they feel are needed.

*[We] get different treatment advice from each health practitioner.*

(ACCHS #5)

*These nurses are a bit no good. They give you Panadol. That’s what they give you … That’s what they are good for. That’s it. I say I have a bad headache.*

(ACCHS #4)

*They sort of do the right thing. Sometimes I ask them about doing an ear check. But they don’t do them.*

(ACCHS #5)

Several community users had ‘worries’ about staff who were unknown to them. These participants had concerns that new staff members who were unfamiliar the medical histories of patients could make the wrong diagnosis and implement the wrong treatment for medical conditions:

*A lot of our old people don’t like going to the clinic, they see new faces. They get worried that they will [be] given the wrong medication. They [new healthcare worker] don’t know our people.*

(ACCHS #5)

#### 3.3.4. Transport

A lack of transport was identified by some community users as a barrier to accessing the main clinic, particularly for after-hours care and specialist consultations. The lack of available transport meant that in some cases, community users were required to walk to and from the clinic during the night and when they were feeling unwell. Transport to specialist consultations out of the community can also be problematic, including missing transportation to appointments and high levels of concern for family members who travel away for healthcare.

*Transportation to clinics (from local homelands), sometimes there are long delays.*

(ACCHS #5)

*When they [patients] get sick at night-time. But they don’t pick the people up. They say you got to find your own way down [to the clinic]. They should pick people up.*

(ACCHS # 4)

Community users from four ACCHSs also reported experiencing difficulties with accessing subsidised patient travel. Some community users told us that their applications to escort family members for medical appointments to larger towns such as Alice Springs and Darwin were declined. When patients did travel without an escort, family members who were left behind in the community experienced challenges contacting patients. Community users reported that they also experience concern about family members’ safety when family members do not have an escort for support when they travel away from their home community for healthcare. They described their fear that family member(s) could come to harm:

*Nephew with leg pain and evacuated to [name of regional centre]. Patient Assisted Travel was requested. But was refused. Community user is concerned when this happens. [They worry about family and if people are going to be alright].*

(ACCHS #5)

*My father had to get to [name of regional centre] for health [care] but they [the clinic] didn’t book a seat for my mother. She is his carer. A lot of bad people go from here to [name of regional centre] and they get the old sick people who are alone to go drinking.*

(ACCHS #3)

### 3.4. Cultivating an Appropriate Workforce for Remote Primary Health Delivery

Although participants were satisfied with many aspects of the healthcare that they received from their local clinic, they provided multiple suggestions to improve the services delivered by healthcare workers. The three main suggestions from community users for cultivating an appropriate workforce were enhancing effective communications, local employment, and services for men delivered by men.

#### 3.4.1. Enhancing Effective Communications

Several community users suggested that communications could be improved by supporting local community members to train as interpreter–translators, thereby creating a pool of interpreter–translators to work at the local clinic. As demonstrated by the quotes below, community users expressed concern for some older community users who may not understand information about medical conditions and medications when explained in English:

*We need more Aboriginal interpreters. A lot of older people leave the clinic. They don’t know the English word to explain their pain. Only language words.*

(ACCHS #4)

*Interpreters [are] needed for better communication between older community members and clinical staff.*

(ACCHS #9)

Community users also acknowledged that access to interpreters supports them to access services that they may be reluctant to access:

*You need a language speaker to encourage us mob to go to the clinic. [To] explain what happens with women’s health check.*

(ACCHS #2)

Some community users acknowledged that family members and local clinic staff and other community members from the local community sometimes assisted with communication during patient–healthcare worker consultations.

#### 3.4.2. Local Employment and Career Pathways

Community users recognise the unique skills and attributes of local people who work in the local clinic, particularly AHPs, and identified these as important to accessing healthcare. Local staff have a unique skill set and knowledge of the local community and issues, as well as the ability to deal appropriately with clinic attendees:

*[There is] no Aboriginal health worker [here]. They could train people from within the community towards these position[s].*

(ACCHS #7)

*They need to train more health workers. Need to get children [youth] involved in health so they can take over from where the older health workers were leading the way.*

(ACCHS #3)

Local employment was identified by community users across all ACCHSs as important for improving the workforce in the local clinic. The benefits of having local staff employed in the clinic would help clinic teams in a variety of ways, including supporting clinic staff to understand local issues and local needs, fostering strong therapeutic relationships between patients and healthcare workers, improving safety for non-local healthcare staff, and supporting communications in the local language.

*More opportunities for young people working in health. What about job experience? Could have young people in the clinic, seeing if they like working there.*

(ACCHS #2)

*We need a cleaner—someone who is from this community. This can be a permanent job for someone. Instead, someone is coming from town to do this job.*

(ACCHS #7)

Across all ACCHSs, male community users suggested more young local men working in clinical and non-clinical roles. ACCHSs have health promotion teams and sport and recreational sections in place. These are all potential options to engage young men in the delivery of healthcare through sport and health (e.g., through job traineeships):

*Recommended need for health promotion teams in remote clinics. These positions may attract more male staff (e.g., sports activities are usually intertwined with health promotion) and provide an entry pathway into the health space for male community members.*

(ACCHS #9)

Local employment was not just discussed in the context of ACCHSs but as employment broadly by all local services. Community users viewed local employment as being important for health and wellbeing:

*We want our young people to be working. Working as a plumber, working at the clinic, working at the shop.*

(ACCHS #4)

Community users also described challenges for local employment that ACCHSs could address. One example is the difficulty of obtaining key documents that are needed for employment such as a Working with Children clearance and not holding a driver’s licence.

#### 3.4.3. Services for Men, Delivered by Men

Both male and female community users reported that they wanted health services for men and for these to be delivered by men. Although a few men told us that they are comfortable seeing female healthcare workers, several men told us that they only go to their local clinic when they are ‘really sick’ or they wait until a male healthcare worker is available. As highlighted by the quotes below, consultation with a female healthcare worker can limit how much male community users share about their condition and contribute to feeling shame and embarrassment:

*Need to have a male doctor. You feel shame when you go and see a female about male health matters. Shame about what is happening to your body. It has been like that for some time … I want to be able to go and see a male doctor.*

(ACCHS #7)

*You can’t tell the [female] nurse about what is going on with your body.*

(ACCHS #1)

*Probably need more men working there. You feel you don’t want to say too much to a woman nurse.*

(ACCHS #3)

There were varying examples provided by community users about what services for men could or should look like. A consistent suggestion was having male healthcare workers including male AHPs, RANs and GPs, who are regularly accessible in the clinic and in specific programs directed to men, such as health promotion.

## 4. Discussion

These findings offer useful insights into how Aboriginal community users view the nature and value of PHC services provided by their local ACCHS in remote and rural locations and the skills and qualities of the local clinic workforce that community users value the most.

Community users of all partnering ACCHSs across Central and Northern Australia want healthcare workers to be appropriately trained for the remote, Aboriginal context. Healthcare workers need advanced clinical skills, culturally safe practice, as well as respect and effective communication skills. These findings align with previous work that identified that community members considered both the cultural and clinical skills of RANs to be critical to delivering quality, appropriate, and accessible healthcare [17]. The qualities of each healthcare worker can affect how and when community users access their local clinic.

High levels of trust and mutual respect between healthcare workers and community users were highly valued by community users. These types of relationships were usually formed by long-term healthcare workers or those regularly returning to one community. In contrast, community users offered many examples of staff who spoke to them disrespectfully. These staff were usually unfamiliar with the community and did not listen to community users. The development of strong relationships with healthcare workers has been identified as one of the factors for sustained engagement by Aboriginal people with their PHC provider [39].

The findings have implications for policy and practice. Building longer-term relationships and both cultural and clinical competence will require stabilisation of the ACCHS workforce. To achieve this, implementing strategies that are shown to work to maximise health workforce retention is needed. This includes preferencing remote and Indigenous student selection into health profession training [40], significant exposure to the remote work environment [41], rural medical placement pathways, and integrated training and employment pathways [42]. Further, community users were also accepting of workforce retention strategies that are implemented by ACCHSs to improve job satisfaction for staff, such as rotating employees into remote communities and other flexible work arrangements [10]. This employment strategy enabled staff to return to the same community to build ongoing relationships with community users, while also allowing staff to attend to family responsibilities regularly. Adequate evaluation of the effectiveness of these retention strategies and incentives is required.

Another central element of the feedback from community users relates to local employment. Consistent with similar studies, the presence of Aboriginal staff was central to ensuring that there was a welcoming environment, a sense of belonging, and a setting where clients felt comfortable and where respect for Aboriginal culture is fostered [39,43].

Community users also recognised the unique cultural knowledge, community engagement, and language skills that Aboriginal staff bring to their role when they are local to the community where the clinic is located [44]. The benefits of healthcare delivery by Aboriginal peoples for Aboriginal peoples is a longstanding recommendation to government by peak bodies, having been reported as early as 1989 in the National Aboriginal Health Strategy [45,46]. The AHW (Aboriginal Health Worker) role (this role transitioned to Aboriginal Health Practitioner (AHP) around 2009 [47]) is recognised throughout Aboriginal communities as critical in efforts to improve Aboriginal health status [46,48]. There have been several initiatives and frameworks to build the capacity of Aboriginal health professionals in the Australian context [38]. Supporting and developing a suitably skilled Aboriginal health workforce leads to improved health outcomes for communities through the provision of culturally safe, person-centred, and holistic care [49]. Local policies providing greater flexibility and varied employment arrangements may increase Aboriginal staff working in ACCHSs by overcoming many of the factors that can have an impact on employment, such as family and caring responsibilities. Overall strategies to attract and support locally based Aboriginal nurses, doctors, and health workers in ACCHSs are beyond the scope of this work, however, demand substantial, long-term commitment, particularly school programs to increase literacy and work-force readiness, school placements in ACCHSs, and the inclusion of subsidised housing in employment packages [50,51].

Although many community users reported accessing their local clinic regularly and receiving high-quality, comprehensive care, there were other persistent barriers to their access to healthcare [52,53,54,55]. Clinic staff with poor communication and interpersonal skills, long wait times, and a lack of confidentiality were particularly problematic when community users provided examples that affected the quality and accessibility of clinic services [17,52]. Improved communication can be achieved by appropriate training of healthcare workers, increased local employment of people with language skills (including as interpreters), and increased availability and use of interpreters.

Consistent with previous reports, other barriers included a lack of transport and limited access to specialist and allied healthcare. Lack of transport, limited specialist and allied health team visits, and long wait times due to workforce shortages in remote areas share an underlying cause of inadequate funding of these services. For example, there is an annual AUD 80 m shortfall of Medicare Benefits Schedule (MBS) funding in the NT [56]. More needs to be done to access MBS in remote clinics, as this money belongs to the community. A key strategy is to ensure that there is capacity for electronic claiming in all clinical information systems. The development and implementation of a national needs-based funding mechanism for remote services that is able to take into account all major Commonwealth and state and territory funding is long overdue. In addition, services should enact policies that ensure specialist and allied health team visits are regular, well-advertised in the community, and have the same staff visiting the same communities.

There was also considerable discussion about men’s health and the need for services delivered for men by male health professionals. Aboriginal men under-utilise PHC services and tend to delay care, often presenting when their illness has significantly progressed [57,58]. There is scant research in this area [59]. Our findings suggest that a major factor why male community users delay going to the clinic is due to the lack of male health professionals, combined with the lack of long-term health professionals who have built trusting relationships with the local community.

Some ACCHSs in this study have implemented effective methods of engaging with their male community users, including clinic design (e.g., separate entrances and waiting rooms), male-only clinics and dedicated spaces for men including mobile health clinics that travel to all communities. Other examples include social and emotional wellbeing teams with Aboriginal staff who facilitate and support access to clinical services for men. If found to be effective, these strategies should be scaled up nationally and be made universally available. Other potentially effective strategies include targeting recruitment of men into clinical and non-clinical roles, such as AHP training and health promotion/sport and recreation activities, respectively, and services ensuring a male AHP or nurse or GP be available at most if not all times.

### Strengths and Limitations of the Study

The fieldwork component of this study occurred largely during the COVID-19 pandemic. During interviews and focus groups, the research team clarified with participants whether the experiences with the health service that they shared were longstanding issues, or if they were new and related to the pandemic. Despite the challenges associated with workforce shortages, limits on travel for short-term staff, as well as prevention of virus transmission as an urgent priority [33], there was generous engagement from ACCHS leadership, staff, and community users across the partnering ACCHSs that were involved in the study. In all but two communities, the research team recruited 20–30 community members across age ranges, genders, and reasons why they access the services. For strong community-led service delivery, ACCHSs have structures to regularly collect input from community users including community boards and feedback mechanisms. The large number of community user participants and their willingness to provide detailed feedback about their experiences at their local clinic reflects the importance of primary care services for remote communities. Ensuring that community users can provide timely feedback through accessible pathways is critical to responding to community suggestions and concerns. Not all clinic sites of partnering ACCHSs were visited. A targeted selection of clinics from each partnering ACCHS was visited in order to collect information from locations that had a high staff turnover and those with generally more stable staffing. Finally, the core qualitative research team were all female. Fostering a gender balance was important for optimising the collection of data. With each ACCHS visit, the research team invested in working with local men as community-based researchers, with male interpreters as well as a male researcher to ensure that community user feedback was collected from male clinic patients.

## 5. Conclusions

This study adds substantially to the previous scant literature by reporting on the perspectives of clinic users on their experiences of healthcare at ACCHS clinics in very remote communities to inform improvements and policy change. This study reaffirms some longstanding issues in remote healthcare delivery. It also highlights new priorities that are important elements of PHC delivered by ACCHSs, affecting access to local PHC clinics. These are complex problems that require multifaceted solutions at the local, national, and international levels. ACCHSs have introduced a number of innovative workforce policies and strategies to attract and retain staff. These include flexible employment arrangements, such as ‘orbiting’ staff who periodically return to the same community and can build relationships with community members over time, as well as job sharing. Some longstanding issues, such as insufficient transportation, communication challenges, infrastructure, and inadequate access to specialist and allied health teams, are symptomatic of deeper issues including inadequate funding of these services, given the burden of disease and distances involved. We need to scale up what we know works, for example prioritising and supporting health professional students from remote areas and Aboriginal students, evaluate new solutions, such as innovative retention strategies, and scale up those strategies that are effective. The provision of quality clinical and culturally appropriate PHC in remote communities, enhanced local employment, improved access to interpreters, and improved access for men are all achievable. If implemented, these strategies will improve, at a lower cost, the health and wellbeing of Aboriginal peoples living in remote Australia.

## Data Availability

The data presented in this study are not publicly available due to privacy restrictions.
